# The first mitogenome of Lesser Whitethroat, *Sylvia curruca blythi* Ticehurst & Whistler, 1933 (Passeriformes: Sylviidae) and its phylogenetic implications for the genus *Sylvia*

**DOI:** 10.1080/23802359.2021.1948367

**Published:** 2021-07-15

**Authors:** Chao Yang, Rong-Jie Yan, Qing-Xiong Wang, Hong Xiao, Xue-Juan Li, Li-Liang Lin, Yan Wang

**Affiliations:** a Shaanxi Institute of Zoology, Xi’an, PR China; b Ecology and Wildlife Conservation and Management Station of Feng County, Baoji, PR China; c School of Life Sciences, Shaanxi Normal University, Xi’an, PR China

**Keywords:** Mitogenome, gene arrangement, phylogeny, *Sylvia*, *Sylvia curruca blythi*

## Abstract

The complete mitogenome of the Lesser Whitethroat, *Sylvia curruca blythi* Ticehurst & Whistler, 1933 was determined, which belongs to Sylviidae, Passeriformes. The mitogenome had a length of 17,959 bp and consisted of 37 genes including 13 PCGs, 2 ribosomal RNAs (rRNA) genes, and 22 transfer RNAs (tRNA) genes. In addition, two control regions (CRs) were also existed in the mitogenome, with Sylvioidea typcial gene arrangement of cytb-trnT-CR1-trnP-nad6-trnE-CR2-trnF-rrnS. Phylogenetic analysis using 37 mitochondrial genes of 17 related species revealed that *S. c. blythi* had a closer relationship with *S. crassirostris*, and the monophyly of *Sylvia* was also recovered. The mitogenome data of *S. c. blythi* would provide useful resources for further studying the evolution of *Sylvia* and the subspecies taxonomic revision of *S. curruca* intraspecific.

The Lesser Whitethroat, *Sylvia curruca blythi* (Passeriformes: Sylviidae), occurs in scrub and hedgerows (Mason 1976). The species is mainly distributed in Beijing, Hebei, Shaanxi, and other places in China (Zheng 2017). The habitats of the species are artificial/terrestrial, forest, savanna, shrubland, and wetlands (inland), with the altitude of 0−2350 m (Birdlife international). The *S. curruca* complex continues to provide challenges for ornithologists and taxonomists (Abdilzadeh et al. 2020). In this study, we determined the mitochondrial genome (mitogenome) sequence of *S. c. blythi*, and analyzed the phylogenetic relationship with other 16 Sylvioidea species.

Naturally dead *S. c. blythi* fledgling from Hongjian Nur of Shaanxi Province, China (39°04′N, 109°53′E), was identified and collected by Q. Wang in 22 May 2013, and the muscle specimen (voucher number BHLY01) was deposited in the animal specimens museum of the Shaanxi Institute of Zoology, Xi’an, Shaanxi Province, China (contacts: Chao Yang, chaoy819@xab.ac.cn).

Total genomic DNA was extracted by DNeasy kit, and sequenced using Illumina HiSeq2000 platform by Genesky Biotechnologies Inc. (Shanghai, China), with the 125 bp paired-end strategy. A total of 15,367,382 paired-end raw reads were yielded. A total of 15,365,225 clean reads were obtained by quality and ambiguity trimed with default parameters using CLC Genomics Workbench version 12.0 The clean data were assembled with MITOBim version 1.9 (Hahn et al. 2013). Finally, a total of 15,303 mitochondrial reads were mapped to the reference mitogenome, *S. atricapilla* (GenBank accession no. NC_010228), gave an average coverage of 126.7X. The mitogenome was annotated using Geneious version 10.1.3 (Kearse et al. 2012) and tRNAscan-SE version 1.21 (Lowe and Eddy 1997).

The complete mitogenome of *S. c. blythi* (GenBank accession no. MG681102) was 17,959 bp in length, including 13 protein-coding genes (PCGs), 2 ribosomal RNAs (rRNAs), 22 transfer RNAs (tRNAs), and plus two control regions (CRs). The gene arrangement of *cytb-trnT-CR1-trnP-nad6-trnE-CR2-trnF-rrnS* was found in the *S. c. blythi* mitogenome, which was similar with that of other Sylvioidea species (Mackiewicz et al. 2019). The overall base composition was as follows: 29.3% A, 32.4% C, 14.8% G, and 23.5% T, with A + T content of 52.8%. The GC-skew was −0.3729, which showed a remarkably C skew and was similar to the mitogenomes of other vertebrates (Saccone et al. 1999). For PCGs, the start codons of the PCGs were all with ATG, except for *cox1* of GTG. The stop codons for translation termination were as follows: TAA (*cox2, atp8, atp6, nad4L*, and *cytb*), TAG (*nad6*), AGG *(nad1* and *cox1*), AGA (*nad5*), incomplete stop codon TA (*nad2* and *nad3*), and incomplete stop codon T (*cox3* and *nad4*). For rRNAs, the two genes of *S. curruca* were 976 bp (*rrnS*) and 1597 bp (*rrnL*) in size, located between *trnF* and *trnFV*, and between *trnV* and *trnL(UUR)*, respectively. For tRNAs, the length were varied from 65 bp in *trnC* to 75 bp in *trnL(UUR)*. Except for *trnS1*, all tRNA sequences could fold into the typical cloverleaf secondary structure. Particularly, the length of *trnS1*’s DHU arm was 0 bp. For CRs, the *CR1* (1106 bp) and *CR2* (1279 bp) were located between *trnT* and *trnP* and between *trnE* and *trnF*, respectively. No repeat units were found in *CR1*, which with 80.8% sequence similarity to *CR2*. No repeat units were also found in *CR2* except for a single base guanine (G) with 30 times tandem repeats during the position 85–114 in 1279 bp (software: tandem repeats finder (TRF) version 4.09; Benson 1999).

The phylogenetic tree was constructed using the newly sequenced mitogenome (*S. c. blythi*) and 16 other available Sylvioidea species, with *Phylloscopus borealoides* (GenBank accession no. MN125373) as a outgroup. The phylogeny employed a dataset containing 37 mitochondrial genes, and were implemented using the maximum-likelihood (ML) method in IQ-TREE version 1.6.12 (Nguyen et al. 2015), with 1000 bootstrap replicates. The topological structure showed that the monophyly of *Sylvia* was recovered, with phylogeny of ((*S. borin*, *S. atricapilla*), (*S. crassirostris*, *S. c. blythi*)) (Figure 1). We also confirmed that *Sylvia* was clustered at the basal position of *Suthora*/*Sinosuthora* clade (Figure 1), which was consistent with that of previous studies (Mackiewicz et al. 2019). This mitogenome would provide important materials for further exploring taxonomic status of *Sylvia* species. Owning to the lack of samples, the morphological variation and taxonomic revision of *S. curruca* subspecies should be further investigated (Olsson et al. 2013; Votier et al. 2016).

**Figure 1.**
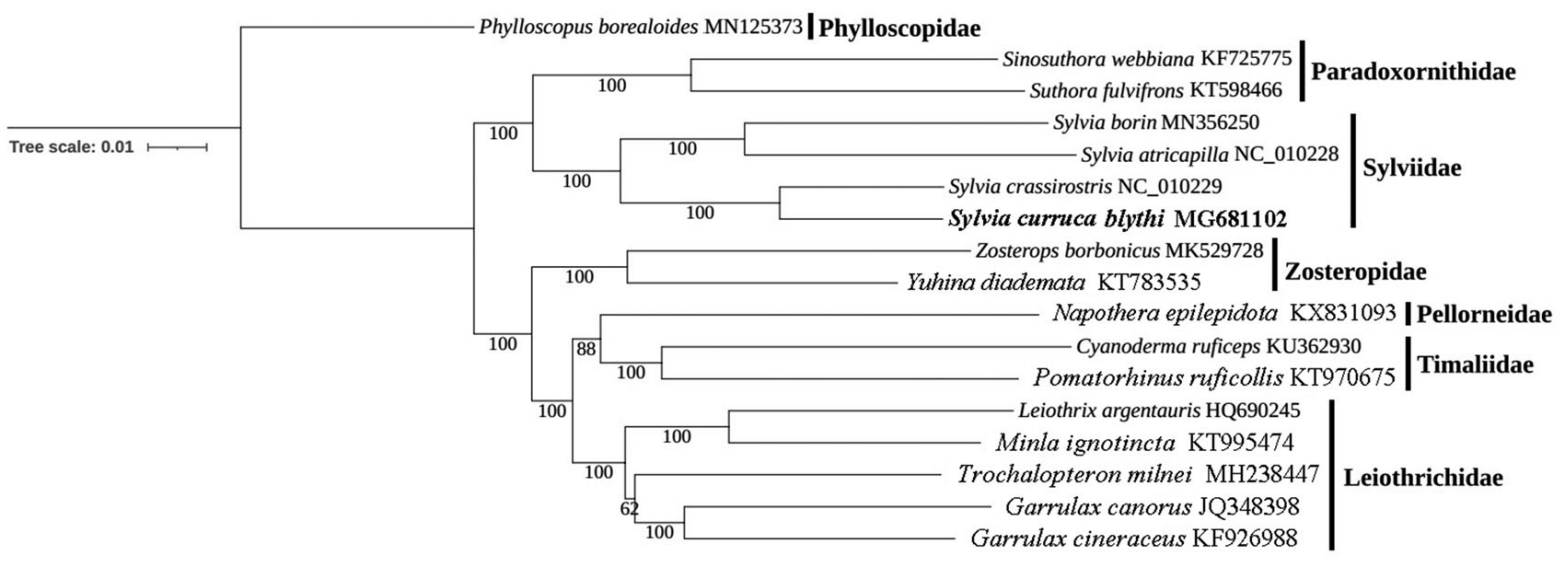
Maximum likelihood tree obtained using IQ-TREE version 1.6.12 with 1000 nonparametric bootstrap replicates. GenBank accession numbers are indicated following species names. Numbers on nodes are bootstrap values.

## Data Availability

The genome sequence data that support the findings of this study are openly available in NCBI GenBank (https://www.ncbi.nlm.nih.gov/) under accession no. MG681102. The associated BioProject, BioSample and SRA numbers are PRJNA721575, SAMN18721429, and SRR14226811, respectively.

## References

[CIT0001] Abdilzadeh R, Aliabadian M, Olsson U. 2020. Molecular assessment of the distribution and taxonomy of the Lesser Whitethroat *Sylvia curruca* complex in Iran, with particular emphasis on the identity of the contentious taxon, *zagrossiensis* Sarudny, 1911. J Ornithol. 161(3):665–676.

[CIT0002] Benson G. 1999. Tandem repeats finder: a program to analyze DNA sequences. Nucleic Acids Res. 27(2):573–580. 9862982 10.1093/nar/27.2.573 PMC148217

[CIT0003] Hahn C, Bachmann L, Chevreux B. 2013. Reconstructing mitochondrial genomes directly from genomic next-generation sequencing reads – a baiting and iterative mapping approach. Nucl Acids Res. 41(13):e129–e129. 23661685 10.1093/nar/gkt371 PMC3711436

[CIT0004] Kearse M, Moir R, Wilson A, Stones-Havas S, Cheung M, Sturrock S, Buxton S, Cooper A, Markowitz S, Duran C, et al. 2012. Geneious Basic: an integrated and extendable desktop software platform for the organization and analysis of sequence data. Bioinformatics. 28(12):1647–1649. 22543367 10.1093/bioinformatics/bts199 PMC3371832

[CIT0005] Lowe TM, Eddy SR. 1997. tRNAscan-SE: a program for improved detection of transfer RNA genes in genomic sequence. Nucleic Acids Res. 25(5):955–964. 9023104 10.1093/nar/25.5.955 PMC146525

[CIT0006] Mackiewicz P, Urantówka AD, Kroczak A, Mackiewicz D. 2019. Resolving phylogenetic relationships within Passeriformes based on mitochondrial genes and inferring the evolution of their mitogenomes in terms of duplications. Genome Biol Evol. 11(10):2824–2849. 31580435 10.1093/gbe/evz209 PMC6795242

[CIT0007] Mason CF. 1976. Breeding biology of the *Sylvia* warblers. Bird Study. 23(3):213–232.

[CIT0008] Nguyen LT, Schmidt HA, von Haeseler A, Minh BQ. 2015. IQ-TREE: a fast and effective stochastic algorithm for estimating maximum-likelihood phylogenies. Mol Biol Evol. 32(1):268–274. 25371430 10.1093/molbev/msu300 PMC4271533

[CIT0009] Olsson U, Leader PJ, Carey GJ, Khan AA, Svensson L, Alström P. 2013. New insights into the intricate taxonomy and phylogeny of the *Sylvia curruca* complex. Mol Phylogenet Evol. 67(1):72–85. 23321212 10.1016/j.ympev.2012.12.023

[CIT0010] Saccone C, De Giorgi C, Gissi C, Pesole G, Reyes A. 1999. Evolutionary genomics in Metazoa: the mitochondrial DNA as a model system. Gene. 238(1):195–209. 10570997 10.1016/s0378-1119(99)00270-x

[CIT0011] Votier SC, Aspinall S, Bearhop S, Bilton D, Newton J, Alström P, Leader P, Carey G, Furnes RW, Olsson U. 2016. Stable isotopes and mtDNA reveal niche segregation but no evidence of intergradation along a habitat gradient in the Lesser Whitethroat complex (*Sylvia curruca*; Passeriformes; Aves). J Ornithol. 157(4):1017–1027.

[CIT0012] Zheng GM. 2017. A checklist on the classification and distribution of the birds of China. 3rd ed. Beijing (China): Science Press.

